# Food allergy‐related bullying in children: Prevalence and psychosocial burden from a mixed‐methods study

**DOI:** 10.1111/pai.70295

**Published:** 2026-02-12

**Authors:** Rita Nocerino, Angelica Esposito, Laura Carucci, Antonio Masino, Caterina Mercuri, Teresa Rea, Silvio Simeone, Roberto Berni Canani

**Affiliations:** ^1^ Department of Translational Medical Science University of Naples “Federico II” Naples Italy; ^2^ NutriTechLab University of Naples “Federico II” Naples Italy; ^3^ Department of Biomedicine and Prevention University of Rome “Tor Vergata” Rome Italy; ^4^ Bachelor of Science in Pediatric Nursing University of Naples “Federico II” Naples Italy; ^5^ Department of Clinical and Experimental Medicine University of Catanzaro Magna Graecia Catanzaro Italy; ^6^ Department of Public Health University of Naples “Federico II” Naples Italy; ^7^ European Laboratory for the Investigation of Food‐Induced Diseases University of Naples “Federico II” Naples Italy; ^8^ Task Force for Microbiome Studies University of Naples “Federico II” Naples Italy

**Keywords:** bullying, children, food allergy, mixed‐methods study, prevention strategies, psychosocial impact, school environment

## Abstract

**Background:**

Food allergy‐related bullying (FARB) is an underrecognized phenomenon that combines the psychosocial harm of peer victimization with the potential for serious physical consequences from allergen exposure. Despite its relevance, data on FARB in Italy are scarce. This study aimed to investigate the prevalence, forms, and lived experiences of FARB among school‐aged children and adolescents with food allergy (FA) using a mixed‐methods approach.

**Methods:**

We conducted an observational cross‐sectional study between June 2023 and April 2024 at a tertiary Pediatric Allergy Unit in Naples, Italy, adopting a sequential explanatory mixed‐method design. Quantitative data on bullying prevalence were collected using the Italian‐adapted Olweus Bully/Victim Questionnaire. Qualitative data were obtained through semi‐structured interviews and analyzed using Interpretative Phenomenological Analysis to explore emotional impact, coping strategies, and perceived support.

**Results:**

Seventy‐three children with FA (mean age 10.6 years; 69.9% male) were included. In the previous 2 months, 35.6% reported at least one bullying episode, and 27.4% identified FA as the specific motive. FARB most frequently involved verbal insults, social exclusion, and rumor spreading, mainly occurring in classrooms and perpetrated by small peer groups. Only 60% of affected children reported the episodes, primarily to parents. Qualitative findings highlighted emotional distress, hypervigilance, social withdrawal, inconsistent adult responses, and a strong desire for greater school awareness and protection.

**Conclusions:**

FARB is a frequent and multifaceted form of victimization with relevant psychosocial and potential physical implications. Preventive strategies should integrate FA management into school‐based anti‐bullying policies, staff training, and inclusive educational interventions.

**Key message**

This study provides the first mixed‐method evidence in Italy showing that just over one quarter of children with food allergy experience bullying directly related to their condition. Food allergy–related bullying is a psychosocial burden as well as a potential threat to physical safety, as it may involve deliberate allergen exposure. By integrating quantitative data with children's own voices, our findings underscore the urgent need for schools to adopt targeted preventive strategies, combining allergy education, staff training, and inclusive policies to safeguard the well‐being, dignity, and participation of affected students.

## 1 INTRODUCTION

Food allergy (FA) is a chronic inflammatory disorder characterized by an abnormal immune response to food antigens, with a growing prevalence that constitutes a public health concern globally^1^, ^2^, ^3^; in fact, according to epidemiological surveys, FA affects approximately 6%–10% of the pediatric population in developed countries.^4^, ^5^, ^6^, ^7^, ^8^, ^9^, ^10^ As demonstrated by the Epidemiology of Pediatric Italian Food Allergy (EPIFA) study, the largest national survey on pediatric FA allergy in Italy, while FA frequently begins in the first years of life, it often extends beyond early childhood, representing a persistent clinical challenge throughout the pediatric age.^2^

The rising incidence, prolonged course, and growing severity of FA throughout childhood have underscored the need not only for sustained clinical vigilance and dietary management, but also for increased attention to the broader psychosocial implications of the condition, as the impact of FA extends far beyond simple dietary limitations, affecting multiple domains of a child's daily functioning and social integration.^11^, ^12^, ^13^

Children with FA are frequently excluded from peer activities, may experience social isolation and are sometimes subject to discriminatory behaviors.^14^, ^15^, ^16^ The persistent risk of inadvertent allergen exposure demands strict avoidance strategies, which can hinder participation in school‐related functions, social events, and cultural practices involving shared meals.^17^

To navigate these daily challenges, affected children often adopt behaviors that set them apart from their peers, such as constant vigilance, adherence to restrictive diets, and the need to be prepared for emergencies.^11^, ^12^, ^18^, ^19^ These burdens may contribute to heightened anxiety levels, diminished independence, and a tendency toward social withdrawal,^11^, ^12^, ^18^, ^19^ with psychosocial consequences that frequently extend to caregivers. Caregivers often experience significant emotional distress, heightened vigilance, and lifestyle restrictions, as well as concerns about their child's social integration and safety, adding a substantial psychosocial and practical burden to family life.^20^, ^21^ Consistently, several studies have demonstrated that FA significantly impairs health‐related quality of life (HRQoL) in both children and their caregivers,^13^, ^22^, ^23^, ^24^ a burden that is further amplified by the constant awareness of the potential for anaphylaxis—a sudden, severe allergic reaction that can be fatal without prompt and appropriate treatment.^23^, ^25^ To mitigate this risk, families must remain perpetually alert and carry emergency medications at all times, contributing to substantial emotional strain.^23^, ^25^

Schools, which serve as crucial environments for development and socialization, may further compound these difficulties.^26^

Emerging evidence suggests that children with FA, who often face stigmatization and inadequate accommodations in educational settings, are increasingly vulnerable to social exclusion and targeted bullying directly linked to their condition,^17^, ^27^ involving mocking, deliberate exclusion, threats of allergen exposure, or even coercive contact with allergenic foods.^20^ In a pivotal study, Lieberman et al.^28^ found that more than one in four children with FA had experienced condition‐related bullying. Subsequent work by Shemesh et al.^20^ reported even higher prevalence rates, with 31.5% of children and 24.7% of parents indicating bullying specifically due to FA. Despite these alarming figures, most research on this topic remains descriptive in nature and lacks standardized methodologies or validated tools for assessing bullying dynamics in the context of FA.

Recent systematic analysis confirms that while food allergy‐related bullying (FARB) affects up to one‐third of allergic children, research in this area remains scarce, methodologically fragmented, and often reliant on parental proxy reporting.^27^ Most studies lack standardized bullying assessment tools, such as the Olweus Bully/Victim Questionnaire (OBVQ), and rarely capture children's firsthand perspectives.^29^, ^30^ Furthermore, there is limited understanding of the emotional repercussions and coping strategies developed by allergic children in response to FARB.

Beyond its psychosocial implications, FARB should also be considered a matter of children's rights. According to the United Nations Convention on the Rights of the Child, every child has the right to protection from all forms of violence and discrimination, as well as to full participation in education and social life (UN General Assembly, 1989).^31^ Framing FARB within this perspective underscores its dual relevance as both a clinical and a societal challenge that requires coordinated action.

Building on these gaps, the present study employs a mixed‐method design aiming to examine the prevalence, typologies, and lived experiences of bullying among children and adolescents diagnosed with FA.

## 2 METHODS

### 2.1 Study design and ethics

This study was designed as an observational, cross‐sectional investigation with a mixed‐methods approach conducted from June 2023 to April 2024 at the Pediatric Allergy Unit of the Maternal and Child Department, University Hospital Federico II in Naples, Italy. A sequential explanatory mixed‐method design was adopted, whereby quantitative data on bullying prevalence were collected first and subsequently complemented by qualitative phenomenological interviews to deepen the understanding of lived experiences. This approach allowed for triangulation of measurable trends with subjective narratives, thereby enhancing the validity and interpretive depth of the findings. Within this sequential explanatory mixed‐methods framework, integration occurred at multiple levels. Quantitative findings informed the selection of participants for the qualitative phase through purposive sampling and guided the focus of the interview questions. Subsequently, qualitative findings were used to contextualize and deepen the interpretation of quantitative results, allowing for triangulation between prevalence data and children's lived experiences. Final integration occurred during the interpretation and discussion phases, where quantitative and qualitative results were examined together to provide a comprehensive understanding of FARB. The study protocol, patient information sheet, informed consent form, and clinical chart were reviewed and approved by the Ethical Committee of the University of Naples Federico II. The study adhered to the Helsinki Declaration (Fortaleza revision, 2013), Good Clinical Practice standards (CPMP/ICH/135/95), and relevant European and Italian data protection regulations.

### 2.2 Participants

Participants were recruited from outpatient pediatric allergy clinics and included children and adolescents with a confirmed diagnosis of FA between 7 and 17 years of age, corresponding to the age range of compulsory and upper secondary education in Italy, ensuring that all respondents were enrolled in formal schooling and shared exposure to the school environment where bullying could occur. Participants were children and adolescents aged 8–17 years.

Eligibility required regular school attendance and the cognitive and linguistic capacity to independently or semi‐independently complete the questionnaires. Diagnoses were based on clinical criteria and confirmed via diagnostic testing including skin prick tests, specific IgE assays, or oral food challenge, according to international guidelines. Exclusion criteria encompassed neurodevelopmental disorders, psychiatric comorbidities, or chronic diseases unrelated to FA that could confound the outcomes or impede participation.

### 2.3 Study outcomes

The primary outcome was the evaluation of the prevalence, forms, and contexts of FARB among children and adolescents with diagnosed FA.

Secondary outcome explored the lived experiences, emotional impact, coping strategies, and perceived support needs of children facing FARB through thematic analysis of open‐ended narratives.

### 2.4 Sampling

The achieved sample size aligns well with the exploratory nature of this study, which aimed to investigate the topic of FARB that remains underexplored both nationally and internationally. The use of a mixed‐methods approach enabled the collection of both quantitative data on prevalence and qualitative insights into lived experiences, enriching the contextual understanding of the phenomenon. For the qualitative component, purposive sampling was applied to select children and adolescents with a confirmed diagnosis of FA who reported experiences of FARB. Selection aimed to capture variability in age, sex, school level, and type of FA. Only participants who completed the questionnaire and met these criteria were invited to take part in the semi‐structured interviews. Recruitment continued until thematic saturation was reached.

The integration of these complementary data sources thus enhanced the interpretive strength and depth of the findings.

### 2.5 Data collection

The research protocol combined structured quantitative instruments with qualitative data collection, enabling an integrative understanding of both measurable and subjective aspects of FARB.

During the initial visit, experienced pediatricians evaluated each subject for eligibility.

After obtaining informed consent from parents or legal guardians, data collection was conducted into three phases. First, socio‐demographic, anamnestic, and clinical information were obtained using a structured form compiled through patient interviews and medical chart review. This form documented variables such as sex, age, birth weight, delivery mode, gestational age, weaning age, personal and familial allergy history, exposure to environmental triggers (smoke, pets, mold), school level, and number of food‐induced anaphylaxis episodes.

Second, the Italian version of OBVQ was administered.^32^ The items explored the frequency of bullying by classmates at school over the past 2 months. One question per type of bullying was included to assess physical, verbal, social, and relational bullying. Responses were rated on a Likert scale from 1 (never happened) to 5 (several times a week). The survey measured general bullying experiences (e.g., “Classmates hit, kicked, or pushed me”) in both children with and without FA. Additionally, according to a previous study, allergic participants were asked about bullying specifically related to their FA. To this end, a modified version of the questionnaire was used, in which each original item was followed by the phrase “because of my food allergy” (e.g., “Classmates hit, kicked, or pushed me because of my food allergy”). The adapted version of the OBVQ had been previously used in an Italian pediatric population and showed acceptable internal consistency (Cronbach's alpha 0.67).^33^ In the present study, this previously employed adaptation was adopted as is, without additional psychometric testing, since the objective was a descriptive exploration of bullying experiences rather than the development or re‐validation of the instrument.

Third, the qualitative component adopted an Interpretative Phenomenological Analysis (IPA) framework, rooted in phenomenology, hermeneutics, and idiography, to explore how participants made sense of their lived experiences.^34^ IPA draws on the philosophical traditions of Husserlian phenomenology, Heideggerian hermeneutics, and an idiographic commitment to the detailed examination of individual cases, aiming to provide both rich descriptions and interpretative insights into meaning‐making processes. This approach is particularly suited to pediatric populations, where eliciting in‐depth narratives benefits from flexibility, multiple prompts, and a conversational style.^35^, ^36^ IPA's idiographic focus on small, purposive samples and depth of inquiry allows for a nuanced understanding of the lived experience of bullying related to FA. Personal accounts were collected through semi‐structured interviews containing multiple open‐ended questions, enabling both rich description and interpretative exploration in a child‐friendly conversational format. Interviews were conducted in a quiet, comfortable, and emotionally safe setting. All sessions were audio‐recorded with consent, transcribed verbatim, and supplemented with field notes documenting non‐verbal cues and contextual observations. A flexible, participant‐oriented interaction was encouraged, allowing children to share their stories in their own words, in alignment with IPA principles that value in‐depth, first‐person accounts and minimize researcher‐imposed structure.^35^, ^36^

### 2.6 Data analysis

Quantitative and qualitative analyses were conducted sequentially, in accordance with the explanatory mixed‐methods design. Quantitative analyses are described first, followed by qualitative analysis of the interview data.

A clinical trial monitor reviewed the clinical forms for completeness, clarity, consistency, and accuracy. All the data were collected anonymously and entered into the study database using a single data entry method by the same researcher. The study database was cleaned according to standard procedures and was locked before statistical analysis by the statistical team.

### 2.7 Quantitative analysis

The Kolmogorov–Smirnov test was used to determine whether continuous variables were normally distributed, in which case they were reported as mean and standard deviation (SD). Continuous variables that were not normally distributed were reported as median and interquartile range (IQR) with minimum and maximum. Categorical variables were reported as the number and proportion of subjects with the characteristic of interest. The *χ* ^2^ test and Fisher's exact test were used for categorical variables. To evaluate the differences between continuous variables, the independent sample *t*‐test or Mann–Whitney *U* test was performed.

The level of significance for all statistical tests was two‐sided, *p* < .05. All analyses were performed using SPSS for Windows (SPSS Inc., version 27.0, Chicago, IL).

### 2.8 Qualitative analysis

Qualitative interview data were analyzed using IPA. The analysis was conducted primarily by one researcher with formal training and experience in qualitative research and IPA. A second researcher independently reviewed and coded a subset of transcripts to enhance analytic credibility. Theme development was discussed iteratively within the research team, and discrepancies were resolved through discussion and consensus, with constant reference to the original transcripts.

The research team involved in the qualitative phase had prior experience in conducting interviews with pediatric populations and in applying phenomenological approaches. Qualitative analysis was performed manually without the use of qualitative data analysis software, in line with the idiographic and interpretative orientation of IPA. Reflexivity was supported through regular team discussions, acknowledgment of researchers' clinical backgrounds, and continuous engagement with the data throughout the analytic process.

Analysis followed the six‐step IPA procedure recommended by Smith et al.^34^ The process began with repeated readings of each transcript to achieve full immersion in the data. During this phase, initial notes were produced, encompassing descriptive, linguistic, and conceptual observations on the text. From these exploratory notes, emergent themes were developed by identifying meaningful patterns, which were then examined to explore connections within each case, allowing the formation of preliminary thematic clusters. The same detailed procedure was subsequently applied to each transcript individually, treating every case on its own terms to preserve idiographic integrity. Finally, patterns across cases were examined, and themes were clustered into superordinate categories that reflected shared meanings while also respecting the unique variations of individual experiences. This iterative, double‐hermeneutic process—where participants make sense of their experiences and the researcher, in turn, interprets that sense‐making—is central to IPA and allows for a layered understanding of the lived experience.^34^, ^36^

The final qualitative results section presents superordinate themes enriched by narrative descriptions and 2–3 verbatim quotations each, to support transparency, credibility, and idiographic depth, ensuring that readers can see both the interpretative process and the participants' voice.^34^

## 3 RESULTS

### 3.1 Quantitative findings

Between November 2023 and March 2024, a total of 80 consecutive pediatric patients with confirmed FA were assessed for eligibility. Seven were excluded due to meeting exclusion criteria—four for functional gastrointestinal disorders and three for neurodevelopmental disorders. Consequently, 73 children were enrolled in the study.

#### 3.1.1 Demographic and clinical characteristics

The majority of participants were male, with a mean age of approximately 10.5 years. Most were born at term, more than half via cesarean section, and three‐quarters had been exclusively breastfed for more than 2 months. Exposure to passive smoking, environmental molds, or household pets was reported in a relevant proportion of the sample, and one in five mothers reported smoking during pregnancy. Skin symptoms were the most common FA‐related manifestation, followed by gastrointestinal and respiratory symptoms. Anaphylaxis was reported in 16.4% of cases. Detailed demographic and clinical characteristics are summarized in Table 1.

**TABLE 1:** Demographic and clinical characteristics of the study population (*n* = 73).

Variable	Mean ± SD/*n* (%)
Sex	Male: 51 (69.9%); Female: 22 (30.1%)
Age at recruitment (months)	127.56 ± 39.27 (range 84–204)
Birth weight (g)	3256.3 ± 485.56
Age at weaning (months)	5.26 ± 1.04
Age at FA diagnosis (months)	63.47 ± 45.38
Gestational age	Term: 68 (93.2%); Preterm: 5 (6.8%)
Mode of delivery	Cesarean: 48 (65.8%); Vaginal: 25 (34.2%)
Exclusive breastfeeding >2 months	55 (75.3%)
Passive smoking exposure	25 (34.2%)
Exposure to environmental molds	15 (20.5%)
Pets at home	20 (27.4%)
Maternal smoking during pregnancy	16 (21.9%)
Positive family history of allergy	24 (32.9%); mean allergic relatives: 1.17 ± 0.64
FA‐related symptoms	Skin: 58 (79.5%) Gastrointestinal: 33 (45.2%) Respiratory: 27 (37.0%)
At least one anaphylaxis episode	12 (16.4%)

#### 3.1.2 OBVQ findings in the overall sample

According to the OBVQ, most participants reported having between two and five good friends in their class. Nearly half expressed a positive perception of school, while the remainder were equally divided between indifference and a negative attitude. In the 2 months preceding the survey, 35.6% of participants reported experiencing at least one episode of bullying, most often in the form of verbal insults, social exclusion, or spreading false rumors. Less frequent but still notable were bullying via mobile phone and damage to personal belongings.

Bullying episodes most often took place in the classroom and were predominantly perpetrated by small groups of classmates. Fewer than one‐quarter of victims disclosed the incidents, most frequently to parents, followed by friends and teachers. Teacher intervention was more frequently reported than peer intervention, which remained low. In some cases, an adult had contacted the school to address the problem. As bystanders, most children reported some degree of empathy toward victims, and nearly one‐third had explained to others what it means to live with FA. Complete findings for the overall sample are presented in Table 2.

**TABLE 2:** Olweus Bully/Victim Questionnaire findings in the overall sample (*n* = 73).

Variable	Category	%
Number of good friends in class	0	—
	1	—
	2–3	28.8
	4–5	35.6
School perception	Like very much	23.3
	Like	24.7
	Indifferent	26.0
	Do not like/Do not like at all	26.0
Bullying in past 2 months	At least 1 episode	35.6
Type of bullying	Offensive name‐calling	50.7
	Social exclusion	42.5
	Spreading false rumors	39.7
	Mobile phone bullying	23.3
	Damage/disappearance of belongings	23.3
	Bullied due to FA	27.4
Setting	Classroom	26.0
Aggressors	Groups of 2–3 classmates	19.2
Reporting of bullying	Any	21.9
	Told parents	76.2
	Told friends	52.4
	Told teachers	42.9
Teacher intervention	Almost always	45.2
Peer intervention	Almost never	34.2
	Occasionally	27.4
Adult contact with school	≥1 time	23.2
Reaction as bystanders	Strong empathy	39.7
	Slight empathy	52.1
Explained FA to others	Yes	28.8

#### 3.1.3 Subgroup analysis: Children bullied due to FA

A subgroup analysis was conducted among the 20 participants who explicitly reported being bullied because of their FA, corresponding to 27.4% of the overall sample. This subgroup had a higher proportion of males and included children from all school levels, with the highest representation in the fourth year of primary school and the second year of lower secondary school. Compared to the overall sample, these children reported a smaller circle of friends, more frequent and persistent bullying episodes, and a greater prevalence of social exclusion and rumor spreading. Mobile phone bullying and damage to personal belongings were also frequent.

The classroom was the most commonly reported setting, and the aggressors were typically small groups, often involving boys. In most cases, bullying lasted from one to 4 weeks, but in one case it continued for 6 months. Reporting bullying rates were higher than in the overall sample, though peer intervention remained limited. Teacher involvement was more frequently reported, and in 40% of cases, an adult had contacted the school more than once to stop the bullying. As bystanders, most children expressed at least some empathy toward victims, and more than one‐third had explained their FA to others. Detailed results for this subgroup are reported in Table 3.

**TABLE 3:** Findings in the subgroup bullied due to food allergy (*n* = 20).

Variable	Category	%
Sex	Male	70.0
	Female	30.0
Mean age (months)	133.2 ± 40.6 (range 84–204)	—
School level	Primary school	50.0
	Lower secondary school	20.0
	Upper secondary school	30.0
School perception	Like	25.0
	Indifferent	35.0
	Do not like	25.0
	Do not like at all	15.0
Number of good friends	None	5.0
	1	35.0
	2–3	30.0
	4–5	30.0
Frequency of bullying	Once/twice in 2 months	75.0
	2–3 times/month	20.0
	Weekly	5.0
Type of bullying	Offensive name‐calling	100.0
	Social exclusion	95.0 (40% weekly)
	Spreading false rumors	95.0
	Mobile phone bullying	75.0 (15% weekly)
	Damage/disappearance of belongings	55.0
	Physical aggression	5.0
	Coercion/threats	5.0
	Due to perceived deficits in ability	25.0
	Racial/religious/disability‐related	0
	Cyberbullying (computer)	0
Setting	Classroom	70.0
Aggressors	Groups of 2–3	55.0
	Groups of 4–9	25.0
	Groups >9	10.0
Aggressor gender	Boys and girls	35.0
	Mainly boys	45.0
Duration of bullying	1–2 weeks	50.0
	1 month	45.0
	6 months	5.0
Reporting of bullying	Any	60.0
	Told parents	80.0
	Told friends	60.0
	Told teachers	46.7
	Told other adult at school	40.0
	Told sibling	6.7
Peer intervention	Almost never	70.0
Teacher intervention	Almost always	35.0
	Occasionally	25.0
Adult contact with school	More than once	40.0
Reaction as bystanders	Strong empathy	30.0
	Slight empathy	65.0
Explained FA to others	Yes	35.0

### 3.2 Qualitative findings

IPA identified five superordinate themes describing children's lived experiences of FARB: (1) Emotional Impact; (2) Defensive Strategies and Coping; (3) Social Devaluation and Bullying Dynamics; (4) Adults' Responses and Trust in the School System; and (5) Hopes for Awareness and Safer Environments.

The IPA revealed five superordinate themes, each illustrating a different dimension of the lived experience of FARB. The themes are presented below with expanded descriptions and representative quotations to highlight both individual nuances and shared meanings.

#### 3.2.1 Theme 1: Emotional impact

Bullying episodes related to FA deeply affected children's emotions. Many described a profound sense of sadness and feeling “wrong” or “inferior,” often internalizing others' negative comments. Others expressed anger and frustration when their condition was mocked or trivialized. Several participants also revealed a sense of resignation, explaining that they tried to ignore the bullying but still felt hurt inside. This theme captures how bullying reshapes a child's self‐perception, eroding their sense of belonging and reinforcing feelings of vulnerability.

The negative comments about my allergy make me feel wrong and different from the others. They say I'm ‘too demanding,’ but I don't think my allergy is a burden for anyone but me. (F, 8 years, multiple food allergies)

It makes me very angry because it's not my fault I have an allergy. I didn't choose this, so why should I feel mortified? (M, 14 years, nut allergy)

When someone doesn't take my allergy seriously or minimizes the problem, I just let it go… I feel resigned to it. (F, 8 years, multiple food allergies)

#### 3.2.2 Theme 2: Defensive strategies and coping

To protect themselves, children adopted different coping strategies. Some chose social withdrawal, avoiding recess or birthday parties to escape humiliation or potential allergen exposure. Others became hypervigilant, closely monitoring their food and surroundings to prevent accidents or pranks. A few oscillated between passive coping—such as ignoring insults—and active coping, like responding with irony or anger. This theme reflects how bullying forces children to live in a constant state of alert, balancing their desire for normality with the need for self‐protection.

At recess, I pretend I'm going to the bathroom so I can eat my snack alone, because I'm afraid they might play a trick with my food. (F, 8 years, multiple food allergies)

When I eat with someone, I always keep my eyes on my plate because I'm afraid they might do something mean. (F, 8 years, multiple food allergies)

When someone makes sarcastic jokes about my allergy, I reply with the same irony, even if deep down it hurts. (M, 14 years, cow's milk allergy)

#### 3.2.3 Theme 3: Social devaluation and bullying dynamics

FA often became a target for ridicule and exclusion, reinforcing stigma and feelings of social inferiority. Some children reported being labeled “contagious” or “too demanding,” while others experienced deliberate allergen threats—allergens placed in their food or on their desk. Social exclusion was also common, with children left out of parties or group activities because of their condition. This theme illustrates how bullying is not just verbal but can involve intentional humiliation, marginalization, and the misuse of food as a tool of power.

They make cow sounds at me because I can't drink milk. I pretend I don't hear, but it makes me feel awful. (M, 7 years, cow's milk allergy)

When I came back from the bathroom, I found a spoon dirty with yogurt on my desk. Nobody wanted to help me clean it. (F, 9 years, cow's milk allergy)

My classmates don't invite me when they go out to eat because they say I'm ‘too demanding.’ It makes me feel really bad and I tend to isolate myself. (F, 8 years, multiple food allergies)

#### 3.2.4 Theme 4: Adults' responses and trust in the school system

Participants described mixed experiences with adults. Some teachers and parents intervened immediately, showing empathy and taking steps to stop the bullying. Others, however, ignored or minimized the problem, leaving children feeling unprotected. This inconsistency eroded trust in the school as a safe environment and made some children hesitant to report incidents. This theme highlights how adult responses can either validate a child's experience and foster safety, or perpetuate their sense of invisibility.

My parents immediately called the school to solve the problem, and some teachers reassured me and really tried to help. (F, 8 years, multiple food allergies)

The teachers ignored the yogurt incident on my desk, pretending nothing happened. (F, 9 years, cow's milk allergy)

I never told the teachers because I didn't want to make it worse, but I also think the school doesn't care about this issue. (M, 14 years, multiple food allergies)

#### 3.2.5 Theme 5: Hopes for awareness and safer environments

Despite their negative experiences, children expressed **constructive hopes** for change. They wanted schools to organize educational activities about food allergies, create visual reminders in classrooms, and ensure better emergency preparedness. Above all, they asked for empathy and equal treatment, wishing that peers and adults would understand the seriousness of their condition without stigma. This theme emphasizes children's desire for a more inclusive and informed environment where their health needs are respected.

Schools should organize courses on food allergies. Teachers should explain that this is serious. (M, 14 years, multiple food allergies)

There should be posters in the classroom about food allergies so everyone can see them and learn. (M, 9 years, nut allergy)

I wish people would know that we are all the same and they shouldn't treat kids with this problem badly but help them. (M, 9 years, nut allergy)

## 4 DISCUSSION

This study is the first in Italy to investigate FARB using a mixed‐methods approach, showing that just over one quarter of children with FA experienced bullying directly related to their condition. While these prevalence rates are consistent with international reports, the present findings add depth by elucidating the mechanisms, contexts, and consequences of FARB through children's lived experiences.^17^, ^27^, ^33^ Interpreting these results within a rights‐based framework further underscores their relevance. According to the United Nations Convention on the Rights of the Child, children are entitled to protection from discrimination and degrading treatment and to equal participation in education.^31^ From this perspective, FARB emerges as both a health concern and a social justice issue, combining emotional harm with the potential for severe physical danger related to allergen exposure.

Beyond its psychosocial burden, FARB entails concrete clinical risks. Deliberate allergen exposure may trigger potentially life‐threatening reactions, including anaphylaxis, as previously documented in pediatric allergy populations.^14^, ^20^, ^25^, ^28^ These findings reinforce that allergy‐related bullying is not merely symbolic but can escalate into medical emergencies, positioning FARB as a patient safety issue that warrants explicit recognition within school health policies.

Children's narratives illustrate how this dual threat shapes emotional well‐being and behavior. Many participants described fear, sadness, anger, and frustration following episodes of mockery, exclusion, or threats, often internalizing negative messages and questioning their self‐worth. These emotional responses align with evidence linking FARB to anxiety, social withdrawal, and reduced self‐esteem.^14^, ^37^ Coping strategies varied, with some children adopting hypervigilance and constant monitoring of food and surroundings, while others withdrew from social situations such as parties or shared meals, further exacerbating isolation.^18^, ^19^ Such behaviors mirror the developmental trajectories described by DunnGalvin and Hourihane, who identify avoidance, heightened vigilance, and adaptive self‐regulation as common responses among children and adolescents with FA; in the context of bullying, these strategies may become intensified as mechanisms to manage both physical and social threat.^38^

Experiences of social devaluation and stigma were prominent across narratives. Children reported being labeled as “too demanding” or “different,” and in some cases deliberately targeted with allergens, contributing to feelings of shame, embarrassment, and marginalization. Adult responses played a pivotal role in shaping children's perceptions of safety. While some teachers intervened promptly and supportively, others minimized or ignored incidents, undermining trust in the school environment and discouraging reporting. These findings are consistent with the systematic review by Newman et al., which highlights how adolescents' beliefs and attitudes toward FA are shaped by peer dynamics and school contexts; limited awareness and inconsistent management practices may reinforce vulnerability and reluctance to seek help.^39^ Despite these challenges, children articulated constructive proposals for change. Suggestions included school‐based allergy education, awareness campaigns, improved emergency preparedness, and inclusive practices aimed at ensuring safety and equal participation. Such recommendations highlight the importance of integrating children's voices into prevention strategies and policy development.^15^, ^33^

In the Italian context, institutional recognition and structured prevention of FARB remain limited. Unlike settings such as the USA and UK, where allergy education and anti‐bullying protocols are increasingly implemented, Italian schools rarely adopt standardized procedures, despite evidence of high prevalence and underestimation by educators.^33^, ^40^ Internationally, school‐based interventions—such as teacher training, student education, and integration of FA protocols into anti‐bullying policies—have shown promise in reducing both bullying and accidental allergen exposure.^15^, ^41^ The absence of systematic responses may contribute to the continued invisibility of FA and the normalization of peer discrimination.

Our findings, derived from a single‐centre Italian cohort, align with international evidence indicating that FARB is a pervasive and underrecognized phenomenon, with prevalence rates ranging from 20% to 35% across Europe and North America.^20^, ^27^, ^28^ Framing FARB as a dual burden is therefore essential: it functions as a psychosocial stressor linked to impaired quality of life and anxiety,^13^, ^18^, ^21^, ^22^, ^25^ while also posing tangible clinical risks due to coercive or accidental allergen exposure.^20^, ^25^ Beyond the individual level, FARB contributes to absenteeism, reduced educational participation, and family stress, amplifying the broader societal burden of pediatric FA.^42^

A rights‐based lens reinforces the urgency of systemic action. International frameworks emphasize that health‐promoting schools must address chronic health conditions to ensure inclusive and equitable learning environments.^31^, ^43^ In our study, inconsistent school responses not only eroded trust but also represented missed opportunities for prevention. Evidence suggests that structured allergy education for staff and students can reduce bullying incidents and improve safety.^33^, ^40^, ^41^, ^44^, ^45^

The psychological toll of FARB extends beyond affected children to their families. From the children's accounts, parents were described as experiencing vicarious stress and feelings of helplessness in response to bullying episodes, echoing findings by Shemesh et al. and Warren et al.^20^, ^21^ Moreover, experiences reported by participants parallel stigma‐related dynamics observed in other chronic pediatric conditions, such as diabetes or epilepsy, where visibility of illness and fear of difference contribute to social exclusion. Situating FARB within this broader stigma framework may inform more inclusive, condition‐agnostic school‐based interventions.

The mixed‐methods design of this study enabled triangulation of quantitative prevalence data with qualitative narratives, revealing dimensions of FARB often missed by surveys alone. While questionnaires quantified the extent of the phenomenon, interviews captured anticipatory anxiety, self‐imposed silence, and perceived stigma, alongside protective factors such as supportive teachers and peers. This integrative approach strengthens the interpretive value of the findings and provides a foundation for future multicentre research.

This study has several strengths, including its mixed‐methods design, the integration of standardized quantitative tools with phenomenological qualitative analysis, and the achievement of thematic saturation across a broad school‐age range. Limitations include the cross‐sectional design, reliance on self‐report, the lack of re‐validation of the FA‐adapted OBVQ in this cohort, and recruitment from a single tertiary centre, which may limit generalizability. Nonetheless, the combined quantitative and qualitative approach enhances the robustness of the conclusions.

In conclusion, FARB is a frequent, multifaceted, and harmful phenomenon with profound implications for the well‐being of children with FA and their families. Addressing it requires urgent, coordinated action encompassing education, policy, and inclusive school practices. Recognizing FARB as both a public health and children's rights priority is essential to safeguarding dignity, safety, and equal participation in school life.

## AUTHOR CONTRIBUTIONS

**Rita Nocerino:** Conceptualization; methodology; investigation; formal analysis; data curation; resources; project administration; writing – original draft; supervision. **Angelica Esposito:** Validation; investigation; visualization. **Laura Carucci:** Investigation; validation; visualization. **Antonio Masino:** Investigation; validation; visualization. **Caterina Mercuri:** Investigation; validation; visualization. **Teresa Rea:** Validation; investigation; supervision. **Silvio Simeone:** Investigation; validation; supervision; writing – review and editing. **Roberto Berni Canani:** Methodology; investigation; visualization; resources; project administration; supervision; writing – original draft.

## CONFLICT OF INTEREST STATEMENT

Roberto Berni Canani has had the following relevant financial relationships with the following manufacturers: Biostime (research grant), Ch. Hansen (research grant, speaker), DBV (research grant), Dr. Schar (research grant), Humana (research grant), iHealth (research grant), Kraft‐Heinz (research grant, speaker), Mead Johnson Nutrition (research grant, speaker), Nestlè (research grant, speaker), Novalac (research grant, speaker), Nutricia (research grant, speaker), Sanofi (research grant, speaker) as part of publicly funded research projects with the support of the Italian Ministry of Health, the Italian Ministry of the University and Research, and the EU. The other authors declared that they have no conflicts of interest.

## ETHICS STATEMENT

The study was approved by the Ethics Committee of the University of Naples Federico II. Parents or legal guardians provided written informed consent; children provided assent.

## DATA SHARING STATEMENT

Deidentified quantitative data (analytic dataset and data dictionary) and statistical code will be made available upon reasonable request to the corresponding author, subject to institutional approval and data‐use agreement.

## Data Availability

Authors have full access to all the data in the study and take responsibility for the integrity of the data and the accuracy of the data analysis.
